# Novel rat Alzheimer's disease models based on AAV-mediated gene transfer to selectively increase hippocampal Aβ levels

**DOI:** 10.1186/1750-1326-2-11

**Published:** 2007-06-09

**Authors:** Patricia A Lawlor, Ross J Bland, Pritam Das, Robert W Price, Vallie Holloway, Lisa Smithson, Bridget L Dicker, Matthew J During, Deborah Young, Todd E Golde

**Affiliations:** 1 Department of Molecular Medicine & Pathology, The University of Auckland, Auckland, New Zealand; 2 Neurologix Research, Inc., Fort Lee, NJ 07024, USA; 3 Department of Neuroscience, Mayo Clinic College of Medicine, Jacksonville, FL 32224, USA; 4 Human Cancer Genetics, The Ohio State University Comprehensive Cancer Center, Columbus, OH 43210, USA

## Abstract

**Background:**

Alzheimer's disease (AD) is characterized by a decline in cognitive function and accumulation of amyloid-β peptide (Aβ) in extracellular plaques. Mutations in amyloid precursor protein (APP) and presenilins alter APP metabolism resulting in accumulation of Aβ42, a peptide essential for the formation of amyloid deposits and proposed to initiate the cascade leading to AD. However, the role of Aβ40, the more prevalent Aβ peptide secreted by cells and a major component of cerebral Aβ deposits, is less clear. In this study, virally-mediated gene transfer was used to selectively increase hippocampal levels of human Aβ42 and Aβ40 in adult Wistar rats, allowing examination of the contribution of each to the cognitive deficits and pathology seen in AD.

**Results:**

Adeno-associated viral (AAV) vectors encoding BRI-Aβ cDNAs were generated resulting in high-level hippocampal expression and secretion of the specific encoded Aβ peptide. As a comparison the effect of AAV-mediated overexpression of APPsw was also examined. Animals were tested for development of learning and memory deficits (open field, Morris water maze, passive avoidance, novel object recognition) three months after infusion of AAV. A range of impairments was found, with the most pronounced deficits observed in animals co-injected with both AAV-BRI-Aβ40 and AAV-BRI-Aβ42. Brain tissue was analyzed by ELISA and immunohistochemistry to quantify levels of detergent soluble and insoluble Aβ peptides. BRI-Aβ42 and the combination of BRI-Aβ40+42 overexpression resulted in elevated levels of detergent-insoluble Aβ. No significant increase in detergent-insoluble Aβ was seen in the rats expressing APPsw or BRI-Aβ40. No pathological features were noted in any rats, except the AAV-BRI-Aβ42 rats which showed focal, amorphous, Thioflavin-negative Aβ42 deposits.

**Conclusion:**

The results show that AAV-mediated gene transfer is a valuable tool to model aspects of AD pathology *in vivo*, and demonstrate that whilst expression of Aβ42 alone is sufficient to initiate Aβ deposition, both Aβ40 and Aβ42 may contribute to cognitive deficits.

## Background

Alzheimer's disease (AD) is a prevalent neurodegenerative disorder characterized by a decline in cognitive function, accumulation of extracellular amyloid-β peptides (Aβ) and intracellular neurofibrillary tangles, and neuronal loss. Numerous AD-linked mutations in amyloid precursor protein (APP) and presenilins (PS) [1-3] alter APP metabolism resulting in accumulation of Aβ42, a 42-amino acid product essential for the formation of parenchymal and vascular amyloid deposits [4], and proposed to initiate the cascade leading to AD [3]. However, the role of Aβ40, the more prevalent Aβ peptide secreted by cells and a major component of deposits in the cerebral vasculature of AD brain [5,6], is less clear.

Current transgenic models of AD utilize overexpression of mutant human APP and PS1 to increase Aβ production and recapitulate AD cognitive deficits and pathologies [7-10]. However, overexpression of APP results not only in increased production of both Aβ40 and Aβ42, but in elevated levels of other APP fragments which can have neuroprotective [11,12], neurotoxic [13] or signaling functions [14] and influence learning and memory [15-18]. In this study we have used adeno-associated viral (AAV) vectors, gene transfer agents that result in stable, long-term transgene expression in neurons [19-21], to target expression of individual Aβ peptides to the hippocampus of adult rats, allowing us to examine the role of each in the pathology and cognitive deficits seen in AD models. The use of viral vectors to overexpress genes implicated in disease pathogenesis has already been successfully implemented to generate non-transgenic rat models of both Parkinson's [22,23] and Huntington's diseases [24,25], with successful transfer of the method to a primate model [26].

AAV1 vectors encoding BRI-Aβ cDNAs, fusions between human Aβ peptides and the BRI protein involved in amyloid deposition in British and Danish familial dementia [27,28], were used to achieve high-level hippocampal expression and secretion of the specific encoded Aβ peptide [4,29] in the absence of APP overexpression. As a comparison we also examined the effect of AAV-mediated overexpression of APPsw (APP containing the Swedish mutations, K670N and M671L). AAV-treated animals were tested for development of cognitive deficits, and brain tissue analyzed for Aβ levels and evidence of extra-cellular Aβ deposition. The results show that AAV-mediated gene transfer is a valuable tool to model aspects of AD pathology *in vivo*, and demonstrate that whilst virally-mediated overexpression of Aβ42 alone is sufficient to initiate plaque deposition, both Aβ40 and Aβ42 levels contribute to the development of cognitive deficits in this model.

## Results

### Infusion of AAV-BRI-Aβ vectors increases hippocampal Aβ expression

**Figure 1:**
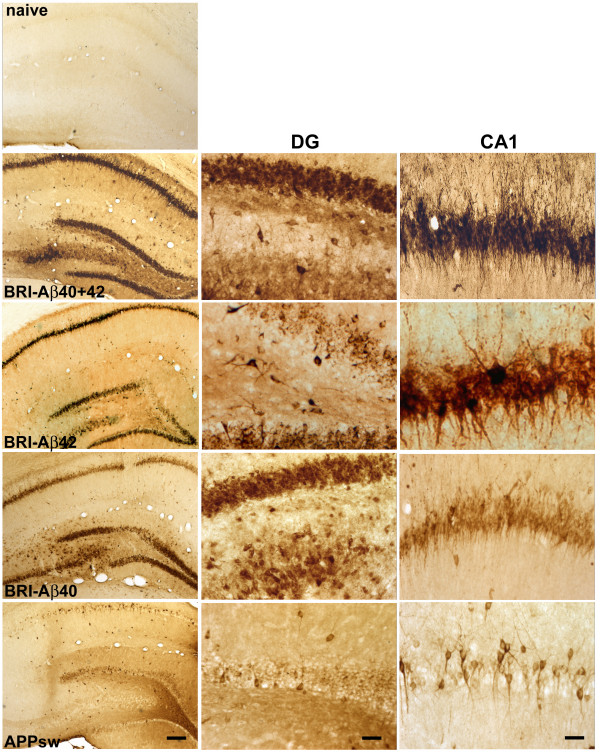
**Transgene expression in rat hippocampus, 3 weeks post-infusion**. A group of test animals (n = 3 per group) was injected with AAV1 vectors and brains analysed for transgene expression (anti-amyloid beta protein, Chemicon, MAB1560, 6E10, specific for human Aβ). No Aβ expression was observed in naïve rat brain. Infusion of AAV1 vectors expressing BRI-Aβ42 and BRI-Aβ40 fusions or APPsw resulted in extensive expression of amyloid β in the dentate gyrus and CA1 fields of the hippocampus, with distinctive expression patterns for each vector. Left panel: low magnification view of unilateral hippocampus, scale bar 250 μm. Middle panel: higher magnification view of dentate gyrus (DG), scale bar 50 μm. Right panel: higher magnification view of CA1, scale bar 50 μm.

AAV1 vectors encoding APPsw and BRI-Aβ42 or BRI-Aβ40 fusion proteins were generated and injected bilaterally into the hippocampus of adult rats, alone and in combination. Brain tissue was examined for presence of transgene expression three weeks later. As seen in Figure 1, infusion of vectors resulted in widespread expression of the transgene in neurons throughout the hippocampus, detectable by immunohistochemistry using an antibody to human Aβ, residues 1–17 (6E10). AAV-BRI-Aβ42, AAV-BRI-Aβ40 and combined AAV-BRI-Aβ40+42 injected brains had extensive transgene expression in the rostral-caudal axis, extending ~2 mm on either side of the injection site, and throughout the layers of the hippocampus. In these brains the granule cell and molecular layers of the dentate gyrus and pyramidal cells of CA1 were transduced (Figure 1). More variable transduction levels were seen in CA2-CA4 subfields and hilar interneurons. In contrast, no transgene expression was observed in naïve (non-injected) rat brain. Given the lack of specific antibodies for the furin-cleaved BRI protein, it is not possible to distinguish whether the staining reflects Aβ or the BRI-Aβ fusion proteins. However, based on ELISA data (see below) and previous studies in transgenic mice, it is clear that the BRI-fusion proteins result in enhanced production of the encoded Aβ peptide [4].

In AAV-APPsw brains, Aβ immunostaining extended as far in the rostral-caudal direction as in BRI-Aβ fusion animals, but staining was not as widespread within the layers of hippocampus. These brains contained much lower expression levels in the granule cells of dentate gyrus and CA1 (Figure 1) than that observed in BRI-Aβ fusion brains, with no positive immunostaining in CA2-4 or hilar interneurons. Distinctive sub-cellular expression patterns were observed with the different vectors – transgene detected following transduction with the BRI-Aβ40 and BRI-Aβ42 vectors filled the cell bodies; transduction obtained with the APPsw vector resulted in sparse transgene expression within the cell soma but expression extended out into the processes.

### Animals treated with AAV vectors encoding BRI-Aβ fusions develop cognitive deficits

**Figure 2:**
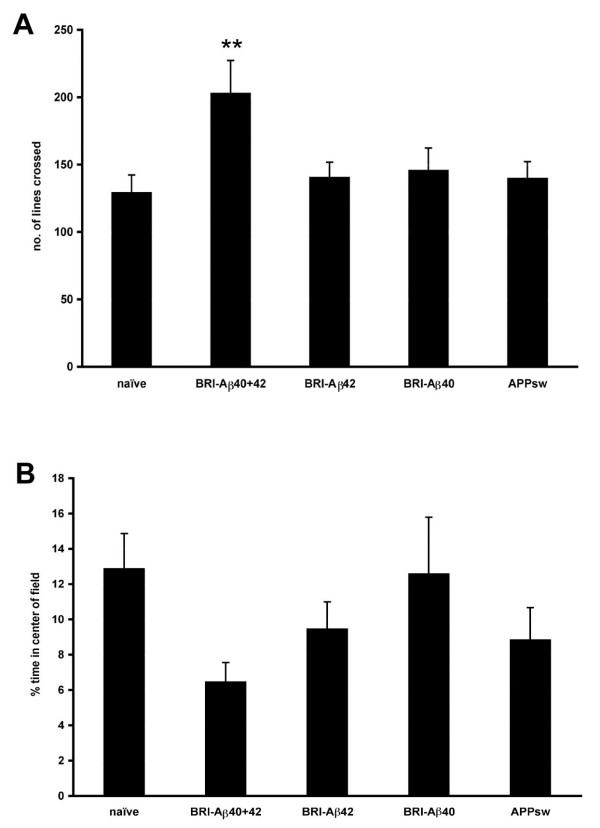
**Behavior in the open field**. 12 rats per group were used for behavioral testing. A. BRI-Aβ40+42 animals crossed significantly more lines than naïve controls or any other treatment group (ANOVA P = 0.027; BRI-Aβ40+42 > naïve, **p < 0.01). B. Although overall there was no significant difference between treatment groups (ANOVA P = 0.24), BRI-Aβ40+42 animals spent the least amount of time in the center of the field.

Once hippocampal transgene expression had been confirmed in a small number of test animals, further animals were injected with AAV vectors and tested for development of cognitive deficits at 3 months post-infusion. In the open field, naive animals crossed an average of ~130 lines in a 5 min period; BRI-Aβ42, BRI-Aβ40 and APPsw animals crossed a comparable number of lines with mean line crosses in these groups of ~140 (Figure 2A). However BRI-Aβ40+42 animals crossed significantly more lines than naïve controls (203 vs 130 lines, p < 0.01) indicating higher baseline locomotor activity than other groups. Naïve rats spent ~13% of their time in the open (center of field) as did the BRI-Aβ40 animals, with a small reduction in time spent in center field in both the BRI-Aβ42 and APPsw groups relative to naive animals. The most marked reduction in time spent in the open however, was in the BRI-Aβ40+42 animals which spent on average only half the amount of time in the open as naive controls (~6% vs 13%) (Figure 2B).

**Figure 3:**
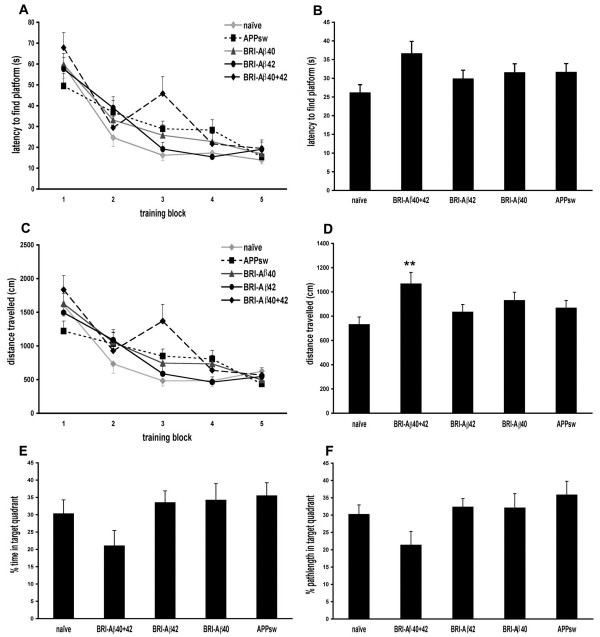
**Morris water maze**. A. Repeated measures ANOVA on latency to find the platform during acquisition shows that overall the difference between treatment groups was not significant (P = 0.12). However, naïve animals took fewer trials to learn the location of the platform than AAV treatment groups, reaching a minimum latency by day 3. BRI-Aβ40+42 animals had a noticeably higher latency on day 3 of acquisition than other groups. B. BRI-Aβ40+42 animals had the highest mean latency over all 20 trials of all treatment groups (ANOVA P = 0.07). C. Repeated measures ANOVA on pathlength to find the platform during acquisition shows that overall the difference between treatment groups was not significant (P = 0.061). D. BRI-Aβ40+42 animals took a significantly longer pathlength to find the platform over all 20 trials (ANOVA P = 0.02; BRI-Aβ40+42 > naïve, ** p < 0.01). E, F. Animals were put through a probe trial 24 hr after the last training trial. BRI-Aβ40+42 animals showed a non-significant reduction in time (ANOVA P = 0.2) and distance (ANOVA P = 0.13) spent in the target quadrant.

In the Morris water maze rats were required to learn the location of the hidden platform over 5 training blocks, with the start point varied between each trial so that the task was difficult enough that differences in performance would be measurable. As seen in Figure 3A, naïve animals learned the location of the platform faster than AAV-treatment groups, with naïve animals reaching a minimum latency of ~18s by day 3 and not improving further beyond that time. All Aβ treatment groups took longer to reach the minimum latency than naïve controls, with the most notable difference between groups on day 3 when BRI-Aβ40+42 animals displayed an increased latency to find the platform. Over all 20 acquisition trials, naive rats had a mean latency of ~26 sec (Figure 3B), with all Aβ treatment groups having longer latencies than this, particularly BRI-Aβ40+42 animals which performed the worst overall with a mean latency over 20 trials of ~37s (ANOVA P = 0.07). Repeated measures ANOVA on pathlength taken to find the platform during the five days of acquisition training confirms the results obtained with latency data. Overall the difference between treatment groups was not significant (P = 0.061) (Figure 3C) but BRI-Aβ40+42 animals took a significantly longer mean pathlength to find the platform over all 20 trials, Figure 3D (ANOVA P = 0.02; BRI-Aβ40+42 > naïve, ** p < 0.01).

Animals were put through a probe trial 24 h after the last training trial to check retention of spatial learning (Figure 3E, F). Naive animals spend ~30% of their time in the target quadrant, as did the BRI-Aβ42, BRI-Aβ40 and APPsw animals. BRI-Aβ40+42 animals spent the least amount of time and distance in the target quadrant of any treatment group (~20%) indicating less retention of platform location than in other groups. Results from the visible platform test demonstrated that visibility was not impaired in treated animals compared to naïve controls, with no difference in latencies to swim to a visible target (data not shown).

**Figure 4:**
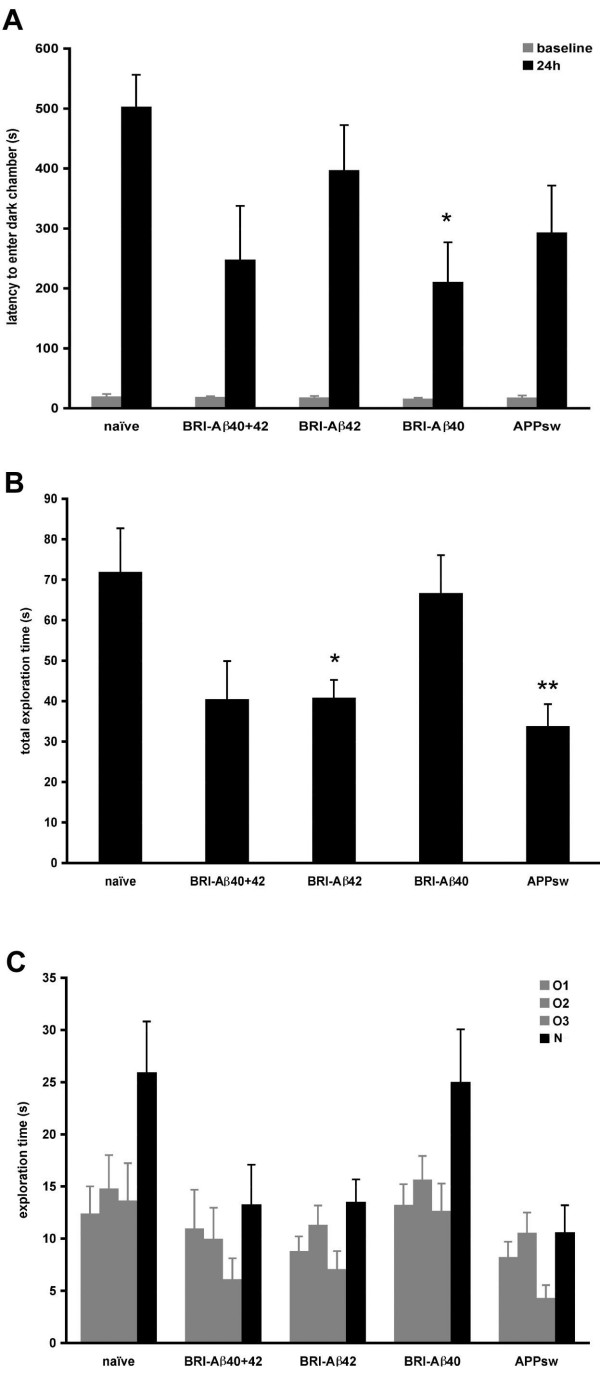
**A. Passive avoidance**. There was no difference in baseline latencies between the treatment groups (ANOVA P = 0.97) but at 24 hr BRI-Aβ40 animals had a reduced latency to re-enter the dark chamber compared to naïve controls (ANOVA P = 0.03; BRI-Aβ40 < naive, *p < 0.05). **B, C. Novel object recognition**. At 3 months post-injection BRI-Aβ42, BRI-Aβ40+42 and APPsw animals spent less total time (O1+O2+O3+N) exploring than naïve controls (B) but still explored the novel object (N) more than the familiar object, O3 (C). Total exploration time: ANOVA P = 0.003; APPsw < naive, p < 0.01, BRI-Aβ42 < naïve, p < 0.05.

In the passive avoidance test there was no difference in baseline latencies to enter the dark chamber between the treatment groups – on being placed in the light chamber for the first time, animals took an average of ~18s to move to explore the dark chamber, indicating no baseline differences in anxiety between treatment groups in this behavioral test (Figure 4A). AAV-BRI-Aβ40 animals had a significantly reduced latency to re-enter the dark chamber 24 hr after receiving a shock as compared to naïve controls (~200s vs 500s, p < 0.05), indicating these animals had an associational learning deficit. BRI-Aβ40+42 animals also had a reduced latency (~240s vs 500s) to enter the dark chamber but high variability between animals meant this did not reach statistical significance. BRI-Aβ42 and APPsw groups had reduced latencies (down by ~20% and 40% respectively) but were not significantly different to controls.

In the novel object recognition test, rats were habituated to the test environment and then given two identical objects to explore (O1, O2). In this first trial all groups spent equal amounts of time exploring both objects, with a ratio O2/O1 of ~1.2 for all groups. Rats were returned to the test chamber 15 min later and presented with one familiar (O3) and one novel (N) object. Animals in all groups recognized and explored the novel object more than the familiar object (with a ratio N/O3 of ~1.9–2.4 for all groups) (Figure 4C). However it is noticeable that BRI-Aβ40+42, BRI-Aβ42, and APPsw animals all spent less total time (O1+O2+O3+N) exploring objects than naïve controls (BRI-Aβ42 < naive, p < 0.05; APPsw < naïve, p < 0.01; Figure 4B) suggesting these animals are less inclined to explore but can still recognize a novel object when encountered. In this behavioral assay BRI-Aβ40 animals were comparable to naïve controls, with similar exploration times and similar ability to discriminate between novel and familiar objects.

Overall, expressing APPsw or either one of the BRI-Aβ fusions alone resulted in distinct cognitive deficits, but interestingly animals expressing both Aβ species exhibited more robust behavioral deficits.

### Animals treated with AAV vectors encoding BRI-Aβ fusions have elevated Aβ levels

**Figure 5:**
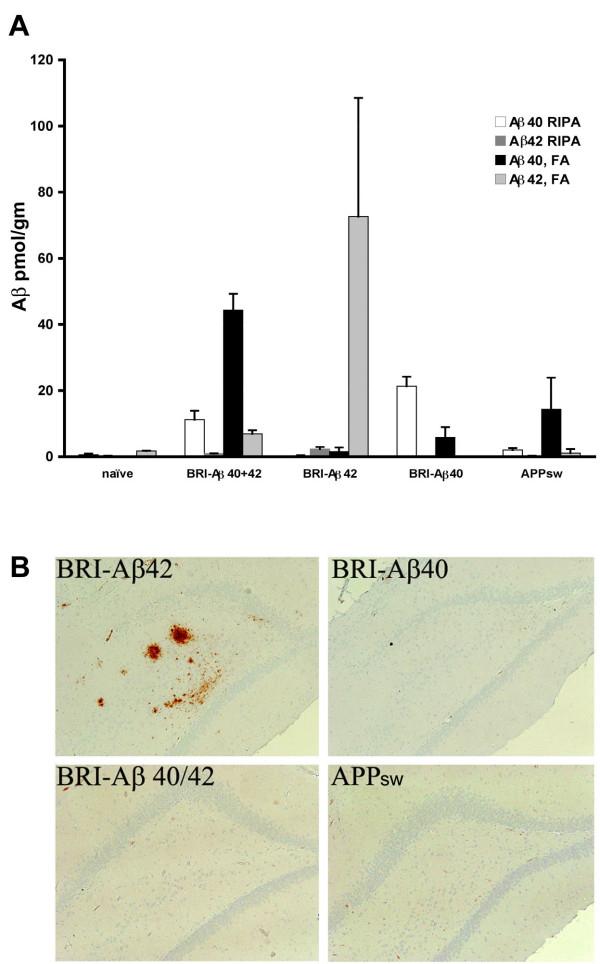
**A. Characterization of Aβ levels in hippocampus by Aβ sandwich ELISA**. 12 rats per treatment were used for biochemical and histological analyses. Naïve rats have very low levels of either Aβ species. AAV-BRI-Aβ40 injected animals display an increase in both RIPA- and FA-soluble Aβ40 at 3 months post-injection. There is a marked increase in FA-soluble (RIPA-insoluble) Aβ42 in AAV-BRI-Aβ42 animals at 3 months. **B. Amyloid pathology in AAV-BRI-Aβ42 animals. **Only BRI-Aβ42 animals showed diffuse plaque-like structures within the hippocampus following immunostaining with pan anti-Aβ 1–16 antibody. These deposits did not stain with thioflavin S or Congo Red (not shown). Magnification 100x.

Animals were killed and the dissected hippocampus examined for levels of RIPA-soluble and RIPA insoluble FA-soluble Aβ peptides (either Aβ40 or Aβ42) by ELISA to assess the levels of Aβ present at the time of behavioral testing (Figure 5A). Naïve rats had negligible levels of Aβ40 or Aβ42 in the hippocampus (all < 2 pmol/gm tissue). AAV-BRI-Aβ40 injected animals had an increase in both RIPA- and FA-soluble Aβ40 but no increase in RIPA- or FA-soluble Aβ42. In AAV-BRI-Aβ42 animals there was a marked increase in FA-soluble Aβ42 to ~72 pmol/gm but only low levels of RIPA-soluble Aβ42 were detected in these brains, suggesting that Aβ42 is accumulating in insoluble deposits. Aβ40 levels (RIPA- or FA-soluble) were not increased above baseline in AAV-BRI-Aβ42 animals suggesting an increase in Aβ42 production did not influence endogenous Aβ40 levels. Animals injected with the combination of BRI-Aβ40+42 vectors had higher levels of RIPA-soluble and FA-soluble Aβ40 than naïve animals and an increase in FA-soluble Aβ42, but no increase in RIPA soluble Aβ42. Interestingly, this level of insoluble Aβ40 is higher than that observed in rats injected with BRIAβ40 alone. AAV-APPsw animals had an increase in the level of FA-soluble Aβ40 (14.3 pmol/gm vs none detected in naïve) but levels of RIPA-soluble Aβ40 and RIPA-soluble and insoluble Aβ42 were comparable to naïve controls.

### Deposition of Aβ plaques was detected only in hippocampus of AAV-BRI-Aβ42 rats

The other hemisphere from each brain was immunostained with anti-Aβ to detect any deposits of Aβ. Only BRI-Aβ42 animals showed "amorphous" plaque-like structures within the hippocampus following immunostaining with pan anti-Aβ 1–16 antibody (Figure 5B). These deposits appeared to be diffuse, confirmed when they did not stain with either thioflavin S or Congo Red (data not shown). Even though there was evidence for significant accumulation of RIPA-insoluble Aβ in the combined BRI-Aβ40+42 expressing rats, these plaques were not seen in the combined treatment group, any other treatment group or the naïve controls. Immunostaining performed on adjacent sections to those with plaque deposition in the BRI-Aβ42 rats showed no evidence of phosphorylated tau, astrogliosis or microgliosis in the vicinity of these diffuse plaques.

## Discussion

To better define the contribution of individual Aβ peptides to the cognitive deficits and amyloid deposition observed in models of AD, virally-mediated gene transfer of BRI-Aβ fusion proteins was used to increase hippocampal levels of individual Aβ species. Use of BRI-Aβ fusions results in enhanced Aβ secretion in the absence of APP overexpression, and distinguishes this approach from overexpression of Aβ minigenes, a strategy that generates high levels of intracellular Aβ but minimal secreted Aβ [30]. Animals injected with AAV1 vectors encoding APPsw, BRI-Aβ40 or BRI-Aβ42, alone and in combination, developed behavioral deficits in a distinct pattern. AAV-APPsw and AAV-BRI-Aβ42 animals had reduced exploration behavior during working memory evaluation but no significant deficits in passive avoidance or acquisition and retention of spatial information. Animals injected with AAV-BRI-Aβ40 alone were impaired in passive avoidance, but were comparable to age-matched naive controls in the working memory task and were not significantly impaired in the Morris water maze. However, animals co-injected with both BRI-Aβ vectors showed the most pronounced behavioral deficits with some impairment in all tests. Despite measurable impairments occurring in all groups, only BRI-Aβ42 animals developed extracellular Aβ deposits. Taken together with the ELISA and histology results, this behavioral data confirms observations from AD transgenic mouse models that measurable behavioral deficits are not dependent on the presence of Aβ plaques. Unexpectedly, the data shows development of more pronounced cognitive deficits when Aβ42 and Aβ40 are co-expressed, and suggests a role for Aβ40, along with Aβ42, in cognitive impairment.

In the current study only AAV-BRI-Aβ42 animals developed extracellular Aβ deposits, located within the hippocampus. These diffuse deposits were immuno-positive for Aβ, but did not stain with thioflavin S or Congo Red and were not associated with astrogliosis or proliferation of microglia, indicating they are "non-cored" diffuse structures. Expression for 9 months enhanced Aβ42 accumulation but still did not result in formation of cored plaques (not shown). Thus, these structures are similar to the diffuse Aβ deposits observed in humans that are primarily composed of Aβ42 and not associated with significant reactive pathology. It is not clear why AAV-BRI-Aβ42 rats do not develop cored plaques. BRI-Aβ42 transgenic mice develop cored plaques in the cerebellum as early as 3 months of age [4], and both diffuse and cored plaques in the forebrain by 12 months of age. However, in those animals brain levels of insoluble Aβ42 were markedly higher than the levels achieved in the current study. In contrast Tg2576 (APP_695_SWE) mice, which have a high ratio of Aβ40 to Aβ42, predominantly develop cored plaques [7,9,31]. Brain-region or species specific factors might regulate Aβ aggregation into diffuse or cored: in humans certain regions of the brain seem more prone to develop diffuse deposits of Aβ, and other factors such as ApoE [32-35] and complement [36,37] may also play a role in driving amyloid formation. In any case, the current study shows that viral delivery of BRI-Aβ42 can foster considerable Aβ accumulation in a relatively short time-frame, and confirms both transgenic Aβ *Drosophila *[38] and transgenic BRI-Aβ mice studies [4] where visible Aβ deposits were obtained only with Aβ42, but not Aβ40, overexpression.

The lack of correlation between presence of plaques and severity of cognitive dysfunction observed in the current study has been noted in a number of AD models [9,39-41]. The current results are consistent with studies in transgenic mice that demonstrate behavioral effects are not correlated with visible plaques, but may correlate better with other Aβ assemblies. Indeed, in Tg2576 mice the appearance of behavioral deficits is associated with the initial occurrence of insoluble Aβ accumulation at a time when no overt plaque formation is noted [9,42-48]. In the current study, overexpression of APPsw resulted in a pattern of deficits similar to that observed in the BRI-Aβ42 group but with no deposition of plaques or detectable increase in insoluble Aβ42, also consistent with data that changes in morphologic markers of synaptic integrity such as dendritic spine density and onset of behavioral deficits can precede a measurable rise in insoluble Aβ42 levels [48].

Data obtained from the combined vector animals suggest some interplay between Aβ40 and Aβ42 levels resulting in enhanced behavioral deficits when the two peptides are co-expressed. The level of insoluble Aβ40 is higher in the BRI-Aβ40+42 animals than in those injected with AAV-BRI-Aβ40 alone. Despite an absence of Aβ deposits that are visible by immunohistochemistry, the presence of SDS-insoluble, formic acid-soluble Aβ indicates that the combined expression of Aβ40 and Aβ42 peptides does lead to insoluble Aβ accumulation, perhaps indicating seeding of Aβ40 deposition by Aβ42. Over-expressing both Aβ40 and Aβ42 in the absence of other APP fragments could result in production of a transient assembly, or a structurally distinct aggregate, that affects behavior to a greater degree than either peptide alone, as seen in a recent study where cognitive dysfunction in Tg2576 mice was linked to formation of a transient soluble assembly [49]. The biochemical analysis of Aβ species described in the current study were conducted prior to these recent reports; thus, the material has been extracted in a manner that would prevent analysis of Aβ * and other oligomeric Aβ species. The behavioral impairments observed in the combined vector treatment group could also be explained by increased anxiety and locomotor behavior – in the open field these animals crossed significantly more lines and spent less time in the open than all other groups. Increased anxiety itself could be indicative of altered hippocampal functioning or damage [50-53].

The exact role of Aβ40 in cognitive impairment is currently unclear. In human patients high plasma concentrations of Aβ40 are associated with an increased risk of dementia [54] and along with Aβ42, Aβ40 is known to impair hippocampal LTP in rats [55-57] and to alter glutamate receptor composition and trafficking and synaptic function [58,59]. However, studies to date on specific effects of Aβ on cognitive function in mammalian brain have relied on non-specific pharmacological intervention to increase Aβ levels [60] or infusion of Aβ peptides directly into the hippocampus or ventricles [61-63] rather than the prolonged and more physiologic secretion strategy adopted here. The pathology of transgenic BRI-Aβ40 and BRI-Aβ42 mice has been characterized [4], but the behavioral phenotype of these animals has not been reported, and to date no behavioral phenotype has been detected (E. McGowan, T. Golde, C. Janus personal communication). A recent study of transgenic *Drosophila *supported a role for both Aβ40 and Aβ42 in age-dependent learning defects [38], with a higher level of Aβ40 than Aβ42 required to affect learning ability. Our data confirm a potential role for both Aβ40 and Aβ42 in altering cognitive impairment that, at least for Aβ40, appears to be dissociable from overt plaque formation. The effects of Aβ40 and Aβ42 in AAV-BRI-Aβ treated rats may reflect alterations in glutamate receptors and synaptic assemblies, and future work will include examination of synaptic markers, NMDA receptor composition and PSD95 levels.

## Conclusion

Overall, the results demonstrate that both Aβ40 and Aβ42, not just Aβ42 alone, may contribute to the development of distinct cognitive deficits in rats as co-expression of Aβ40 and Aβ42 produced a more robust behavioral phenotype then expression of either Aβ peptide alone. The lack of correlation between severity of behavioral deficits and presence of Aβ deposits confirms previous studies of mouse transgenic models in which cognitive deficits precede visible Aβ deposition, but are associated with the accumulation of detergent insoluble Aβ. These results demonstrate that increasing Aβ levels by AAV-mediated gene transfer, allowing spatial and temporal regulation of specific Aβ species, is a valuable tool to study AD pathogenesis.

## Methods

### Study outline

AAV1 vectors encoding APPsw, BRI-Aβ42 and BRI-Aβ40 were generated and injected into the hippocampus of adult rats. Transgene expression was confirmed immunohistochemically in a group of test animals (n = 3 per vector). The effects of long-term over-expression of BRI-Aβ42, BRI-Aβ40, combined BRI-Aβ42 + BRI-Aβ40, or APPsw in the rat hippocampus were assessed, with animals tested 3 months post-infusion for development of cognitive deficits and brain tissue examined for Aβ levels and presence of AD pathology (n = 12 per group).

### Expression constructs

cDNAs for APPsw (gift of D Selkoe) and BRI-Aβ fusions [29] were sub-cloned into an AAV expression plasmid under the control of a CBA (chicken beta-actin) promoter and containing a SAR (scaffold attachment region) element, WPRE (woodchuck hepatitis virus post-transcriptional-regulatory element), and bovine growth hormone polyadenylation signal flanked by AAV2 inverted terminal repeats (ITRs).

### Generation of AAV vectors

HEK293 cells were co-transfected with AAV and helper plasmids using standard CaPO_4 _transfection. Cells were harvested 60 hr following transfection, and AAV1 vectors purified from the cell lysate by ultracentrifugation through an iodixanol density gradient followed by Q column purification, then concentrated and dialysed against PBS [64,65]. Vectors were titered using real-time PCR (ABI Prism 7700) and all vector stocks diluted to 1 × 10^13 ^genomes/ml.

### Infusion of vectors

Animal studies were approved by The University of Auckland Animal Ethics Committee, and rats supplied by the Animal Resources Unit, The University of Auckland. 250–300 g male Wistar rats were used for all studies. Animals were anaesthetized with sodium pentobarbitone (Nembutal, Virbac Laboratories, 90 mg/kg, i.p.) and placed in a Kopf stereotaxic frame. AAV1 vectors were infused bilaterally into the hippocampus at the following stereotaxic co-ordinates: flat skull – anterior-posterior (AP) -4.0 mm, lateral (L) 2.1 mm, and ventral (V) 4.5 mm from skull surface, bregma = zero). 3 μl vector (3 × 10^10 ^genomes) was infused into each side at 70 nl/min. Animals receiving both BRI-Aβ vectors still received a total of 3 × 10^10 ^genomes in 3 μl (1.5 × 10^10 ^genomes of each vector) into each side.

### Behavioral testing

All animals were pre-handled for 7 days prior to testing and tests were carried out in the order described. All tests were conducted by an experimenter blinded to the treatments.

#### Open field

Movement in an open field was used to assess whether AAV treatments had an effect on locomotor ability or anxiety, either of which may affect learning and memory behaviors. Rats were placed in the center of a 1.8 m circular field divided into segments and allowed to move freely for 5 min. The number of lines crossed and amount of time spent within 20 cm of the perimeter were recorded as measures of locomotor ability and anxiety. Time not spent at the perimeter was defined as time spent "center field".

#### Morris water maze

This test was used as a measure of spatial learning – the rat must learn the location of a hidden platform by referring to visual cues placed around the room. The platform location was kept constant throughout training but the starting point varied pseudorandomly between trials, with all four start points used in each training block to ensure all rats had to swim the same distance each day. The water maze consists of a circular pool (1.8 m diameter and 0.6 m height) filled to a depth of 27 cm with 26 ± 1°C water. The escape platform is 17 cm in diameter and submerged 2 cm below the surface of the water. A small amount (5 ml) of non-toxic black textile dye was added to the water to camouflage the platform. Visual cues (black cardboard shapes on white curtains) were placed around the room and remained constant throughout testing, removed only for the visible platform test at the end of the run.

The test procedure consisted of three parts; acquisition training, a probe (retention) trial, and visible platform training. Acquisition training took place over 5 days, with each rat given four trials per day. A trial consisted of allowing the rat 90 sec to find the platform (if not located in this time then the animal was guided to the platform by the experimenter). The rat remained on the platform for 30 sec before being returned to the home cage for 60 sec, when the next trial began. The latency and pathlength to find the hidden platform were recorded for each trial using Watermaze 4.0 Software.

On day six animals underwent a retention (probe) trial – the platform was removed from the pool (visual cues still visible) and the animal allowed to swim for 30 sec. Time spent in the quadrant of the pool that previously held the platform (target quadrant) was measured. On day seven animals underwent visible platform training – visual cues were removed from the wall and the platform placed in a different quadrant, raised above the waterline so it was clearly visible to the rat. The time taken by the rat to swim to the visible platform was recorded. This was to ensure that any observed deficits in learning were not merely the result of poor eyesight.

#### Passive avoidance

This was used as a test of associational learning. Equipment consisted of two identical chambers (one well-lit, the other dark) connected via a guillotine door. Rats were placed in the light chamber and (baseline) time to enter the dark chamber recorded. The door was closed and the animal received a small electrical shock (1.0 mA for 3 sec) whilst in the dark chamber and 10s later was returned to the home cage. Animals were returned to the light chamber 24 hr later and latency to enter the dark chamber was recorded to a maximum of 600s.

#### Novel object recognition

This working memory test is based on the rat's natural propensity to explore novel objects. The test chamber consisted of a perspex box (dimensions 85 × 60 × 50 cm). Rats were given two days to habituate to the test environment – on days one and two animals were placed in the test chamber for 5 min with no objects to explore. On day three the rat was placed in the test chamber now containing two identical objects (O1 and O2; objects never encountered before and of no natural significance). The rat was placed equidistant from the two identical objects and the time spent exploring each object (nose within 2 cm of object) recorded for 3 min. The rat was returned to the test chamber 15 min later and given one familiar (O3) and one novel (N) object to explore. Time spent exploring each object was recorded over 3 min.

### Statistical analyses

Behavioral data was analyzed using ANOVA (repeated measures where appropriate) with Dunnett's post-hoc analysis.

### Tissue collection

Test rats (used to confirm transgene expression) were euthanised with pentobarbitone and perfused transcardially with 60 ml saline followed by 60 ml 4% paraformaldehyde in 0.1 M phosphate buffer (PFA). Brain tissue was post-fixed for 24 h in 4% PFA, cryoprotected in increasing concentrations (10, 20, 30%) of sucrose in PBS and cut into 40 μm free-floating sections using a cryostat. All other rats were sacrificed one week after the conclusion of behavioral testing – animals were euthanised with pentobarbitone and brains removed and dissected longitudinally. One hemibrain was post-fixed in 4% PFA and used for immunohistochemical and histological analyses; the other half was frozen immediately on dry ice and used for Aβ ELISAs.

### Immunohistochemistry

Sections from transgene expression brains were immunostained with anti-Aβ 1–17 (Chemicon MAB1560, 6E10, specific to human Aβ 1–17) according to the following protocol. Sections were washed in 1 × PBS containing 0.2% Triton (PBS-T), and incubated in 1% H_2_O_2 _in 50% methanol for 30 min to bind endogenous peroxidase present in the tissue. Sections were washed extensively in 1 × PBS-T, then treated with 70% formic acid for 30 min and again washed in PBS-T. 200 μl of primary antibody diluted 1:1500 in immunobuffer (1 × PBS-T containing 1% normal goat serum, 0.4 mg/ml methiolate) was applied overnight at room temperature on a rocking table. The following day sections were washed in 1 × PBS-T and incubated in 200 μl of biotinylated anti-mouse (Sigma; diluted 1:250 in immunobuffer) for 3 hr at room temperature. Following further washes in 1 × PBS-T, sections were incubated in 200 μl ExtrAvidin Peroxidase (Sigma; diluted 1:250 in immunobuffer) for 2 hr at room temperature. Sections were washed in PBS-T and antibody binding was visualised using 3', 3-diaminobenzidine (DAB; Sigma, St. Louis, MO) at 0.5 mg/ml DAB in 0.1 M phosphate buffer with 0.01% H_2_O_2_.

Hemibrains from long-term animals were post-fixed in 4% PFA and stained for Aβ plaques as described previously [66]. Paraffin sections (5 μm) were pretreated with 80% formic acid (FA) for 5 minutes, boiled in water using a rice steam cooker, washed, and immersed in 0.3% H_2_O_2 _for 30 minutes to block intrinsic peroxidase activity. Sections were then incubated with 2% normal goat serum in PBS for 1 hour, with 33.1.1 (Pan Aβ 1–16 mAb) at 1 μg/ml dilution overnight, and then with HRP-conjugated goat anti-mouse secondary mAb (1:500 dilution; Amersham Biosciences) for 1 hour. Sections were washed in PBS, and immunoreactivity was visualized by DAB according to the manufacturer's specifications (ABC system; Vector Laboratories). Adjacent sections were stained with 4% thioflavin-S for 10 minutes. Additional sections were immunostained for activated microglia using anti-Iba1 (1:3000; Wako Chemicals); astrocytes using anti-GFAP (1:1000, Chemicon); phosphorylated tau with CP13 (1:100) and PHF-1 (1:100; kindly provided by Dr Peter Davies, Albert Einstein School of Medicine, Bronx, NY).

### Aβ ELISA

Rat hippocampal and cerebellar tissue was homogenized in radio-immunoprecipitation assay (RIPA) buffer and ultracentrifuged to separate RIPA-soluble from insoluble fractions. RIPA-insoluble proteins were extracted using 70% FA, neutralized with Tris base buffer and samples diluted (from 10–5000-fold). Extracted Aβ was then measured using a sandwich ELISA system as described previously [66] – Aβ42 capture with mAb 2.1.3 (mAb40.2,) and detection with HRP-conjugated mAb Ab9 (human Aβ 1–16 specific); Aβ40 capture with mAb Ab9 and detection with HRP-conjugated mAb 13.1.1 (mAβ40.1).

## Competing interests

The author(s) declare that they have no competing interests.

## Authors' contributions

PL carried out surgeries, behavioral testing, data analysis and drafted the manuscript. RB participated in study concept and design, generated constructs and AAV vectors and helped draft manuscript. PD, RB, VH, and LS ran ELISAs and histological analyses of brain tissue. BD performed behavioral assays. MD and DY participated in concept, design and manuscript preparation. TG participated in concept, design, data analysis and manuscript preparation. All authors read and approved the final manuscript.
